# Use of Clinical Preventive Service and Related Factors in Middle-Aged Postmenopausal Women in Korea

**DOI:** 10.3390/healthcare8020083

**Published:** 2020-04-01

**Authors:** Kisook Kim

**Affiliations:** College of Nursing, Chung-Ang University, Heuksoekro 84, Dongjak gu, Seoul 06974, Korea; kiskim@cau.ac.kr; Tel.: +82-2-820-5723

**Keywords:** postmenopause, middle age, health behavior, health promotion

## Abstract

Postmenopausal women experiencing health transitions can improve health-related quality of life through clinical health service use. The aim of this study was to investigate the factors affecting clinical preventive service use, focusing on a multi-dimensional approach among middle-aged postmenopausal women. This descriptive study is a secondary analysis of the seventh Korea National Health and Nutrition Examination Survey (KNHANESVII-1) in 2016. Among the 8150 participants, our analysis included 771 naturally menopausal women aged 40–65. National health insurance (OR = 1.659, 95% CI = 1.080–2.550), private health insurance (OR = 2.877, 95% CI = 1.665–4.971), needs for health service (OR = 2.363, 95% CI = 1.332–4.195), cardiovascular disease (OR = 1.570, 95% CI = 1.009–2.445), hospital admission (OR = 3.054, 95% CI = 1.298–7.184), smoking (OR = 0.262, 95% CI = 0.144–0.477), drinking (OR = 0.573, 95% CI = 0.335–0.979), and depression (OR = 0.535, 95% CI = 0.340–0.841) were associated with clinical preventive service use among middle-aged postmenopausal women. To promote clinical preventive service use among postmenopausal women, policies promoting health behavior expansion should be introduced and should consider the predictive variables revealed by this study.

## 1. Introduction

Preventive health behaviors are actions taken to prevent diseases or detect them early; examples of such behaviors include a balanced diet, exercise, and health screening. Among various types of health behaviors, preventive health behaviors include activities such as vaccination and health screening [1]. Clinical preventive service can identify and prevent disease in people who have unhealthy lifestyles and motivate them to change behavior [2], and regular clinical preventive services are effective ways to promote health and avoid disease [3]. Clinical preventive services also help to reduce the number of hospital admission days, outpatient days, and health-related costs; accordingly, clinical preventive services have been applied through various health policies [4,5]. Especially for the middle aged—the 50 to 64 age group—maintaining a healthy lifestyle with regularly recommended screenings and vaccinations is important for healthy aging and preventing the leading causes of death and disability [5]. Currently, health policy is undergoing a paradigm shift from acute- and treatment-focused care to preventive health care [1]. Based on this trend, preventive health behavior is considered important at all ages, but especially, the period of menopause is psychologically difficult and changes women’s lifestyles in various domains, increasing vulnerability to physical and mental health problems [6,7,8]. 

Menopause is a normal degenerative transition associated with aging and fertility decline [6]. Middle-aged women, most of whom are postmenopausal, may experience a variety of disabilities that can lead to death or impair quality of life [9]. The leading causes of death among women in developed countries include cardiovascular disease, including ischemic heart disease and stroke; cancer; chronic obstructive pulmonary disease; and diabetes [9]. Nevertheless, a significant proportion of these diseases can be prevented, and 34.6% of deaths from chronic diseases are preventable in Korea [10]. Accordingly, there have been several studies on the relationships between metabolic syndrome, cardiovascular disease, and obesity among menopausal and middle-aged women, that have revealed high incidence [11,12,13]. Clinical preventive services, such as routine disease screening and scheduled immunizations, are key to reducing death and disability and improving the nation’s health [14], and increasing the number of people who take advantage of and have access to clinical preventive services continues to be a major public health challenge, not only cerebrovascular accident (CVA) [14,15].

Despite the importance of clinical preventive services for middle age health, until now, associated factors for the promotion of preventive health behaviors have been reported for specific groups, such as the elderly [1,16], people with intellectual disabilities [17], and mental illness patients [18]. Moreover, there is a dearth of published research regarding postmenopausal women experiencing health transitions and who need to improve health-related quality of life through clinical preventive services.

Therefore, in this study, we aimed to identify the factors associated with clinical preventive service use among middle aged postmenopausal women by a multidimensional approach including the health environment, current health status, health behavior and psychological health-related factors. The findings of this study can inform the development of customized disease prevention and intervention strategies for postmenopausal women.

## 2. Methods

### 2.1. Study Design

This descriptive, correlational, cross-sectional study aimed to investigate the factors affecting clinical preventive service use among middle-aged postmenopausal women.

### 2.2. Study Population and Data Source

This study used data from the seventh (2016) Korea National Health and Nutrition Examination Survey (KNHANESVII-1), a nationally representative survey undertaken by the Korea Center for Disease Control and Prevention (KCDC). The KNHANESVII-1 consists of a composite sample of 4416 households in 192 survey groups nationwide, including 8150 persons. Each participant provided written informed consent prior to participation. Our analysis included the 771 women in the survey who were aged 40–65 and were naturally menopausal. We excluded women with artificial menopause (e.g., secondary to hormone therapy, chemotherapy, or surgery).

### 2.3. Measures

#### 2.3.1. Clinical Preventive Service Use

In this study, clinical preventive services were defined as influenza vaccination in the preceding year, health screening in the preceding two years, cancer screening in the preceding two years, and oral check-ups in the previous one year according to the national examination schedule. The definition of a health screening included a private health check-up, an industrial routine health check-up, and a health check-up conducted by the National Health Insurance Corporation. In this study, the clinical preventive service inclusion criteria referred to previous studies [1,19]. The clinical preventive service use group included cases where one or more clinical preventive service was used (*n* = 662) by the individual, with others classified as the non-use group (*n* = 108).

#### 2.3.2. Included Variables

The demographic characteristics of the subjects were analyzed by age, education, number of co-habiting family members, occupation status, and monthly household income. 

Menopause age, postmenopausal period, body mass index (BMI), days of serious illness (one year), current chronic disease and cancer, hospital admission during the previous year, and current activity limits were included as physical health-related factors. Chronic diseases, including CVD (hypertension, dyslipidemia, stroke, myocardial infarction, and angina), osteo-joint related disease (osteoarthritis, rheumatoid arthritis, and osteoporosis), respiratory diseases (asthma and pulmonary tuberculosis), diabetes and kidney failure, and hepatic disease (hepatitis B, hepatitis C, and liver cirrhosis) were included as variables. Cancer was also included as a variable, including gastric cancer, liver cancer, colon cancer, breast cancer, cervical cancer, lung cancer, thyroid cancer, and other cancers. The current activity limit was determined by yes or no responses to the question “Are you currently limited in your daily life and social activities due to health problems or physical or mental disabilities?”. Smoking, alcohol consumption, days of walking (during one week, for at least 10 minutes), and days of strength exercise (during one week) were included as health-behavioral factors. Subjective health status, stress, and depression (PHQ-9) [20] were included as psychological health-related factors. Subjective health status was determined by Likert-type scale responses to “What do you think about your usual health?”, from 1 (very good) to 5 (very bad). Stress was determined by responses to the question “How often do you feel stress in your daily life?” from 1 (very much) to 4 (almost not feeling). Additionally, urban vs. rural residence, unmet required health services, type of national health insurance, and type of private health insurance were included as health environment factors.

### 2.4. Ethical Consideration

The original data of the KNHANES utilized in this study are in compliance with Korean personal information protection law and statistical law, and only the data that cannot be estimated from the survey data are provided. The researchers applied for the data on the homepage with the necessary information and used the data after receiving KCDC approval (https://knhanes.cdc.go.kr/).

### 2.5. Statistical Analysis

The data were analyzed using SPSS Statistics for Windows version 23.0 (IBM Corp., Armonk, NY, USA). The National Health and Nutrition Survey was conducted using two-stage stratified cluster sampling, and the stratified cluster extraction and weighting were applied and analyzed. The general characteristics and main variables of the subjects were analyzed using descriptive statistics. The clinical preventive service use, related variables, and general characteristics of the subjects were analyzed using t-tests and Χ^2^ tests. Logistic regression analyses were performed to examine the associations between clinical preventive service use and related factors. 

### 2.6. Data Statement

The data used in this study can be obtained by e-mail free of charge by applying for raw materials at the Korea Center for Disease and Prevention (KCDC) website: https://knhanes.cdc.go.kr/knhanes/sub03/sub03_02_02.do.

## 3. Results

### 3.1. Demographic Characteristics and Health Environment Factors

The characteristics of the participants included in this study are shown in Table 1. Of the 771 subjects, 663 (85.9%) were in the use group and 108 (14.0%) were in the non-use group.

Among the demographic characteristics, mean age was significantly higher for the use group (t = −3.551, *p* < 0.001). In the case of health environment factors, there were statistically significant differences in terms of the national health insurance type, the private health insurance type, and unmet required health services.

Among the health environment factors, national health insurance type (Χ^2^ = 16.132, *p* = 0.001), private health insurance (Χ^2^ = 9.682, *p* = 0.003), and the prevalence of unmet required health service needs (Χ^2^ = 9.350, *p* = 0.008) were different by usage. There were no statistically significant differences in education, monthly household income, living with family members, occupation, or residence areas.

### 3.2. Current Health Status, Health Behavior, and Psychological Health-Related Factors

The differences in current health status-related factors were statistically significant in terms of the following variables: postmenopausal period, days of illness, chronic disease, CVD, osteo-joint disease, and hospital admissions. The mean postmenopausal period was longer in the use group (t = −2.866, *p* = 0.004), and the number of days of illness of the non-use group was higher (t = 3.744, *p* < 0.001). CVD (X^2^ = 7.421, *p* < 0.001), diabetes or kidney disease (X^2^ = 3.973, *p* = 0.046), and hospital admissions (X^2^ = 6.774, *p* = 0.009) in the use group were also higher. Hospital admissions were also more frequent in the use group (X^2^ = 6.774, *p* = 0.009), while differences in menopause age, BMI, other chronic diseases, and activity limits were not statistically significant.

Smoking (X^2^ = 24.445, *p* < 0.001) and drinking (X^2^ = 5.582, *p* = 0.018) were significantly different by usage. There was no statistically significant difference between the groups in terms of days of walking and days of strength exercise per week.

Regarding psychological health-related factors, the depression score was higher in the non-use group (t = 2.792, *p* = 0.005). Subjective health status and stress did not show statistically significant differences between groups (Table 2).

### 3.3. Factors Associated with Preventive Health Behaviors

The association between clinical preventive service use and related factors’ odds ratios (OR) and confidence intervals (CI) was estimated after adjusting for the significant demographic characteristic, age.

As a result of discriminant analysis and mean comparative analysis, variables (*p* < 0.05) that were found to differ according to clinical preventive service use were subject to regression analysis. Included variables were national health insurance type, private health insurance, unmet health service requirements, days of illness, CVD, diabetes or kidney disease, hospital admissions, smoking, alcohol drinking, and depression. Because the median value of the ‘days of illness’ was 7.0 (mean: 22.54, SD: 48.47), the cutoff point was set at 7. In addition, the cutoff point of diagnosed depression was set at 5, because 7.3% (56 participants) had a score of 10 or higher in this study [17].

Regression analysis revealed that national health insurance (salary based) (OR = 1.659, 95% CI = 1.080–2.550), private health insurance (OR = 2.877, 95% CI = 1.665–4.971), and needs for health services (OR = 2.363, 95% CI = 1.332–4.195) were health environment factors associated with clinical preventive service use among middle-aged postmenopausal women. In addition, CVA (OR = 1.570, 95% CI = 1.009–2.445) and hospital admission (OR = 3.054, 95% CI = 1.298–7.184) were statistically significant health status factors. Smoking (OR = 0.262, 95% CI = 0.144–0.477), drinking (OR = 0.573, 95% CI = 0.335–0.979), and depression (OR = 0.535, 95% CI = 0.340–0.841) were associated with clinical preventive service use (Table 3).

## 4. Discussion

Preventive health behaviors began to gain attention when the Ottawa Charter for Health Promotion was adopted at the International Conference on Health Promotion held in Canada in 1986; in Korea, the Health Promotion Act of 1995 marked their increasing importance [20,21].

This study showed that 86.0% of postmenopausal women used one or more clinical preventive services. Additionally, it has been shown to affect, in the multidimensional domain among health environment factors, current health status factors, health behavior factors, and psychological health related factors. These findings are partially consistent with the age, level of education, marital status, income, health insurance, and subjective health status variables reported in previous studies in relation to the clinical preventive service use of middle-aged women in Korea and the U.S. [19].

For elderly Korean Americans, education appeared to be associated with clinical preventive service use [22], but the results of this study did not confirm this. In various previous studies, income has shown associations with clinical preventive service use [1,17,19,22]; however, this association was not statistically significant in the present study. 

Participants with national health insurance coverage had a higher clinical preventive service use rate than those with occupational health insurance, such as that paid depending on salary (OR 1.699, 95% CI = 1.080–2.550). However, due to the characteristics of the Korean health care system, 97.2% of Koreans are covered by national health insurance, and 72.4% are covered by schemes that depend on salary; therefore, finding a statistically significant result was not expected. Due to the wide range of national health coverage and the provision of state-supported clinical preventive health services, it is considered to be that there was no difference between the city and rural service rates.

In this study, it was found that private health insurance holders accessed clinical preventive services 2.252 times more. Private health insurance, on the other hand, has been associated with clinical preventive service use in various studies [22,23]. This suggests that private health insurance holders with self-purchased plans that require additional payments practice clinical preventive service more frequently than holders of mandatory national health insurance. Nevertheless, in some studies, health insurance gain has been associated with an increase in clinical preventive service use, such as vaccination and cancer screening, but not with significant changes in other health behaviors [24]. Moreover, the fulfillment of health service requirements, which is a variable related to the promotion of clinical preventive service use, could be pursued by improving education and satisfaction [25]. In this study, the participants whose health service requirements were met had a clinical preventive service use odds ratio of 2.363. Thus, improvements to the existing health care environment, as well as publicity and educational campaigns, will be needed. 

As a result of this study, the clinical preventive service use odds ratio for days of illness was 0.529 times lower, and the odds ratios for CVA, diabetes mellitus (DM) and kidney disease, and hospital admission were higher: 1.810 to 2.949. Menopausal symptoms have been associated with hospitalization frequency due to illness [26], and healthy lifestyle has been associated with decreased CVD-related hospitalization in postmenopausal women [27]. Clinical preventive service use in the present study was associated with the postmenopausal period. These results are likely to be related to the increase in the postmenopausal age, the increase in the number of free vaccinations for elderly people (over 65 years of age) in Korea, and some health policy outcomes for elderly health promotion [1]. However, in the case of postmenopausal women among the vulnerable elderly, as determined by educational level and ethnic minority status, the health behavior execution rate is low [28]. Therefore, efforts to improve clinical preventive service use according to these vulnerable characteristics will be needed. Moreover, since the age of menopause is 50–52 years, perimenopause, menopause, and postmenopause comprise half or a third of women’s lives, especially in developing countries [8]. Therefore, it will be necessary to expand clinical preventive service use by early postmenopausal women.

CVD, which is the number one cause of death among Korean women, has a particularly high prevalence after the age of 40, when menopause begins [29]. This result showed that subjects with CVD had a better usage of clinical preventive services; however, the link between having a family history of CVD and health-related behavioral change was inconsistent [30]. Although the treatment of CVD is similar in both genders, adequate risk stratification may be limited in women compared to in men [31]. Therefore, clinical preventive service use is more likely to prevent or reduce the risk of CVD than of other diseases, and it is necessary to continue to expand clinical preventive service policies for postmenopausal women.

Clinical preventive service use is not consistent with drinking and smoking in the elderly group. The influenza vaccination rate has been shown to be higher among non-smokers and non-drinkers, with health-screening frequency being higher among smokers and drinkers, and a higher cancer screening rate among smokers [1]. However, the results of this study suggest that non-smoking is a clear factor associated with clinical preventive service use, which is consistent with a previous study in which smoking, inactivity, and obesity were shown to be important factors in preventable diseases due to behavioral changes [32]. 

In this study, we could not confirm a direct relationship between clinical preventive service use and activity, but health-related changes in lifestyle, such as activity, have been reported to be related to CVD risk reduction among postmenopausal women [12,32]. In particular, the predictive variables of clinical preventive service use by the postmenopausal women derived from this study were smoking, CVD, the length of the postmenopausal period, and days of illness.

The most common multiple lifelong health issues among women are unlike those among men, or they have no effect on men at all. While women’s health issues are important at all stages of life, the health concerns of middle-aged and elderly women are not stressed by scientists and policy makers [9]. Expenditure on preventive health care services has been shown to be negatively associated with health care costs; in addition, investment in preventive health activities reduces health care-related costs [4]. The prevention of chronic diseases and disabilities is a key aspect of improving women’s quality of life and ensuring an active role throughout aging for middle-aged and elderly women in society [9]. 

This study has the following strengths. In particular, it uses the National Health and Nutrition Survey data, which are representative of the Korean middle-aged female population, to identify factors influencing clinical preventive service use in postmenopausal middle-aged women. This provided fundamental data to inform the development of a customized health care intervention and of disease prevention strategies for postmenopausal middle-aged women. Moreover, there have been a number of studies focusing on the changing of single health behaviors, and there are not many studies dealing with multiple clinical preventive service use.

This study also has some limitations. As a cross-sectional study, it could not reach conclusions on clinical preventive service use in individual women, and causality could not be decisively determined. In addition, because this study involved secondary data analysis, it did not identify the relationship with various other variables affecting clinical preventive service use, due to limited data. In addition, clinical preventive service use may include a wide range of behaviors other than health checks, but only 2-year health check experiences, according to the national health screening schedule in this study, were included. Therefore, there is a limit to expanding the results of this study to clinical preventive service use.

## Figures and Tables

**Table 1:** Demographic characteristics and health environmental factors according to clinical preventive service use.

Variables		Use (*n* = 663)	Non-Use (*n* = 108)	t or X^2^	*p*
		Mean (S.D.)/*n*(%)			
**Demographic Characteristics**					
Age		58.18 (4.59)	56.50 (4.45)	−3.551	<0.001
Education	Elementary School	188 (87.0)	28 (13.0)	3.501	0.321
	Middle School	154 (89.5)	18 (10.5)		
	High School	213 (83.9)	41 (16.1)		
	≥College	108 (83.7)	21 (16.3)		
Monthly Household Income (USD)		4132.0 (3228.2)	3967.6 (3137.7)	−0.490	0.625
Min–Max	170–15,000				
Living with Family Members		2.73 (1.09)	2.72 (1.11)	−0.029	0.977
Occupation	Have	378	56	1.006	0.347
	None	285	52		
**Health Environmental Factors**					
Residence Area	City	527 (85.4)	90 (14.6)	0.859	0.436
	Rural	136 (88.3)	18 (11.7)		
National Health Insurance Type	A type	225 (82.7)	47 (17.3)	16.132 *	0.001
	B type	423 (89.1)	52 (10.9)		
	C type	14 (63.6)	8 (36.4)		
	No	1 (50.0)	1 (50.0)		
Private Health Insurance	Yes	591 (87.4)	85 (12.6)	9.682	0.003
	No	71 (75.5)	23 (24.5)		
Required Health Service	Unmet	56 (74.7)	19 (25.3)	9.350 *	0.008
	Met	592 (87.4)	85 (12.6)		
	No Required Service	15 (78.9)	4 (21.1)		

PHB: Preventive Health Behaviors; * Fisher’s exact test; A type: Paid depending on property and income; B type: Paid depending on salary; C type: Covered by government due to low income.

**Table 2:** Current health status, health behavior, and psychological health-related factors according to clinical preventive service use.

Variables		Use	Non-Use		
		Mean (S.D.)/*n*(%)		t or X^2^	*p*
**Current Health Status Factors**					
Menopause Age		50.41 (3.38)	50.22 (3.17)	−0.528	0.598
Postmenopausal Period (year)		7.78 (5.08)	6.28 (4.78)	−2.866	0.004
BMI (kg/m^2^)		24.16 (3.37)	24.15 (3.24)	−0.040	0.968
Days of Illness (days)		16.74 (39.19)	55.61 (83.11)	3.744	<0.001
Chronic Disease	Cardiovascular	308 (89.8)	35 (10.2)	7.421	0.006
	No	355 (82.9)	73 (17.1)		
	Osteo-joint	228 (89.4)	27 (10.6)	3.699	0.054
	No	435 (84.3)	81 (15.7)		
	Respiratory	43 (86.0)	7 (14.0)	0.000	1.000
	No	620 (86.0)	101 (14.0)		
	DM & Kidney	80 (93.0)	6 (7.0)	3.973	0.046
	No	583 (85.1)	102 (14.9)		
	Hepatic	16 (94.1)	1 (5.9)	0.953*	0.492
	No	647 (85.8)	107 (15.6)		
	Cancer	44 (91.7)	4 (8.3)	1.368	0.242
	No	619 (85.6)	104 (14.1)		
Hospital Admissions (1 year)	Yes	98 (94.2)	6 (5.8)	6.774	0.009
	No	565 (84.7)	102 (15.3)		
Activity Limits	Yes	61 (88.4)	8 (11.6)	0.366	0.545
	No	602 (85.8)	100 (14.2)		
**Health Behavior Related Factors**					
Smoking	Yes	35 (63.6)	20 (36.4)	24.445	<0.001
	No	626 (87.7)	88 (12.3)		
Alcohol Drinking	Yes	149 (81.0)	35 (19.0)	5.582	0.018
	No	240 (88.9)	30 (11.1)		
Days of Walking/1 week		3.64 (2.02)	3.50 (1.90)	−0.627	0.531
Days of Strength Exercise/1 week		1.57 (1.36)	1.56 (1.44)	−0.037	0.970
**Psychological Health Related Factors**					
Subjective Health Status		3.02 (0.88)	2.99 (0.90)	−0.300	0.764
Stress		2.85 (0.75)	2.87 (0.73)	0.304	0.761
Depression		2.77 (3.78)	3.94 (5.24)	2.791	0.005

**Table 3:** Factors associated with clinical preventive service use among postmenopausal women.

Variables	OR	Adjusted OR	95% CI		*p*
Health Environment Factors					
National Health Insurance (Salary Based)	1.699	1.659	1.080	2.550	0.021
Private Health Insurance (Yes)	2.252	2.877	1.665	4.971	<0.001
Required Health Service (Met)	2.363	2.363	1.332	4.195	0.003
Current Health Status Factors					
Days of Illness (Over 7 Days)	0.529	0.482	0.194	1.195	0.115
CVA (Yes)	1.810	1.570	1.009	2.445	0.046
DM & Kidney Disease (Yes)	2.333	2.080	0.878	4.925	0.096
Hospital Admission (Yes)	2.949	3.054	1.298	7.184	0.011
Health Behavior Related Factors					
Smoking (Yes)	0.246	0.262	0.144	0.477	<0.001
Drinking (Yes)	0.532	0.573	0.335	0.979	0.041
Psychological Health Related Factors					
Depression (PHQ ≥ 5)	0.562	0.535	0.340	0.841	0.007
